# Delivery of dietary messages for type 2 diabetic patients by dental practitioners: A scoping review protocol

**DOI:** 10.1002/hsr2.70131

**Published:** 2024-10-08

**Authors:** Kailey Paterson, Kay Franks, Janet Wallace, Dileep Sharma

**Affiliations:** ^1^ School of Health Sciences, College of Health Medicine and Wellbeing The University of Newcastle Ourimbah New South Wales Australia

**Keywords:** counseling, dental, diabetes, diet, health education

## Abstract

**Objective:**

The objective of this scoping review is to identify and understand the available evidence on the delivery of dietary messages to patients with type 2 diabetes in a dental setting. The outcome of a scoping review in this area will inform the development of a clinical intervention for dietary counseling at the chairside.

**Introduction:**

Diabetics are at a higher risk for developing periodontal disease, and the severity of periodontal disease can impact the ability to control glucose levels. Considering the prevalence of diabetes within the community, dental practitioners are well placed to provide dietary messages to support this cohort during the management of periodontal disease.

**Inclusion Criteria:**

Studies that consider the population affected by type 2 diabetes and a dietary intervention in the context of a dental setting will be included.

**Methods:**

The databases selected for sources of studies are MEDLINE, EMBASE, CINAHL and SCOPUS. The scoping review will be conducted in accordance with the Joanna Briggs Institute methodology for scoping reviews. Only English language studies are eligible for inclusion in this review. Terms relating to dietary advice, diabetes, dental practitioner and health education will be used to search for related studies. Screening based on abstract, and titles will be followed by full text screening with results supplied in PRISMA‐SCR diagram. A data extraction tool will be used to chart the details of selected studies then presented in a venn diagram and word map along with a narrative synthesis of results.

## 1 INTRODUCTION

Diabetes is a widespread health issue affecting 529 million worldwide, type 2 diabetes mellitus (T2DM) accounts for 96% of cases.^1^, ^2^, ^3^ Poor dietary habit is considered one of the a major factors in the prevalence of diabetes worldwide.^3^ Notably, patients with T2DM will need to alter their food intake to stabilize blood glucose levels (BGL) and maintain healthy weight to reduce the risk of complications.^2^, ^4^ It is generally agreed that successful management of diabetes benefits from regular reinforcement of diabetes education, including dietary messages.^5^, ^6^

T2DM is associated with extensive inflammation and compromised healing, with a bidirectional relationship suggested with other inflammatory conditions such as obesity and periodontal disease.^7^, ^8^, ^9^, ^10^ The pathological changes associated with T2DM hyperglycemia or insulin resistance initiates an inflammatory cascade. This immune response to the inflammatory load is overreactive, particularly in the presence of destructive periodontal diseases.^7^

Periodontal disease (PD) is a group of inflammatory conditions affecting the gingival tissues and the surrounding periodontium and includes gingivitis and periodontitis. According to the Australian National Study of Adult Oral Health 2017–18, 29% of adults over 15 years old had gingivitis and 30.1% had moderate or severe periodontitis.^11^ While gingivitis is reversible with treatment, periodontitis is only manageable and requires life‐long support.^12^ Plaque (a bacterial biofilm) is the primary cause of gingivitis, however the impaired healing in diabetic patients and elevated risk for periodontitis warrants further education about this relationship. Lalla et al.^13^ found that participants with diabetes had higher plaque scores and more attachment loss from a young age. The inflammatory response incited by PD leads to the loss of supporting structure and eventual tooth loss, in cases of periodontitis.^2^, ^10^ A comparison of the National Study of Adult Oral Health from 2004–06 and 2017–18 found that, unlike other dental diseases, the population of Australians experiencing PD had increased.^14^ Additionally, severe PD is associated with a higher risk of T2DM diagnosis. This is mediated by the immune response to plaque bacteria resulting in a persistent stimulation of the systemic immune response, contributing to poor BGL control and insulin resistance.^15^ Obesity, T2DM and PD appear to be linked by this low‐grade inflammation theory, correlating all three conditions.^7^ Wu et al.^16^ suggested that periodontitis should be a cue to investigate poor glycaemic control in T2DM patients and highlight potential undiagnosed cases. Furthermore, PD treatment has been shown to positively impact BGLs.^17^ Thus, as periodontitis outcomes are correlated to achieving a glycaemic stability,^9^ dietary messages can play an important role in the prevention and management of these chronic conditions.

Although links between T2DM and PD have been well documented,^2^, ^9^, ^16^ the preventive role of dental practitioner (in diet) is an underutilised skill.^18^ In Australia, the dental practitioners scope of practice for providing dietary advice is defined as “Analysis of and advice on a patient's diet, including alcohol consumption, to correct any dietary imbalances or deficiencies that may contribute to oral diseases.”^19^ However, previous studies suggest that the dietary advice provided by dental practitioners is very limited, if at all provided.^18^ While the potential to expand this skillset under the guidelines is evident, time constraints and lack of training act as barriers in providing dietary advice.^18^ Thus, educational resources targeting dental practitioners are needed to build and utilize this skillset. Dietary education for T2DM patients has the potential to be included within the existing treatment and maintenance framework of PD. This provides the diabetic cohort with an additional source of support for managing lifestyle changes associated with T2DM, and the benefit of improving periodontal outcomes.

As diabetes is a known risk factor for PD and increases the risk, progression and severity of the periodontal disease,^20^ it is important that messaging about PD is delivered through a targeted approach in conjunction with dietary information. This highlights the potential to involve dental practitioners in multidisciplinary management of diabetes and associated complications. The detection of T2DM can provide an opportunity for practitioners to encourage more regular maintenance visits due to the risk of PD progression. Incorporating dietary advice within PD maintenance visits could potentially improve the success of behavioral change^21^ by minimizing access barriers and creating another cue to action.

### 1.1 Rationale

It is within dental practitioners’ scope of practice in Australia to provide dietary advice as an integral part of managing dental diseases. However, this is focused on management of dental caries and dental erosion, that leads to destruction of hard tissues via bacterial metabolic acid and external/internal acidic exposure respectively.^22^ The extensively researched links between PD and T2DM, and the inclusion of diabetes in the updated PD classification system^12^ highlights that this is an area that requires development. Given the high level of T2DM and PD cases worldwide and the intersection of these two groups, it is evident that more support is required for this cohort. Although there appears to be no reviews collating data relating to psychological intervention of dietary counseling by dental practitioners, there is evidence that “psychological models of behavior provide a framework for the design of interventions to enhance oral health‐related behavior.”^23^ A scoping review will assist with conceptualizing current knowledge and identifying gaps within the literature. The process will also identify the current and emerging trends in research around the delivery of dietary messaging in the oral health space, specifically relating to the diabetic population accessing dental care.

### 1.2 Objectives

The objective of this scoping review is to map out existing literature relating to delivery of dietary messages to diabetic patients within a dental setting. The evidence being sought is related to the methodology of these studies on the delivery of messages. It is not to deduce the most successful outcomes, but rather to gather information about what methods and/or behavioral systems have been used to target dietary behaviors in this population. The information gathered may include type of dietary information delivered to this population, clinician delivering the dietary messages within the dental team, clinical settings, mode of messages delivered or recommended mode and/or the outcomes in a dental setting. The outcome of this review will be used to guide the development of a clinical intervention based on psychologically informed framework that can be delivered at the chairside by a dental practitioner as part of a PhD project conducted by author (KP). Preliminary searches have not revealed any scoping reviews within the interest area of this review.

## 2 METHODOLOGY

The authors will conduct the scoping review in accordance with the Joanna Briggs Institute (JBI) methodology for scoping review.^24^ Additionally, this scoping review protocol has been developed with the framework provided by JBI manual for evidence synthesis.

### 2.1 Inclusion/exclusion criteria

The population, concept and context will be used to guide selecting sources for inclusion within the scoping review. The participants refer to the population of T2DM, the concept refers to the dietary intervention and the context refers to the dental setting. Reviews, and non‐peer reviewed sources such as conference abstracts, articles, government documents, and patient educational documents will not be included in the scoping review.

### 2.2 Participants

Any studies involving participants with T2DM, pre‐diabetes or medically diagnosed elevated BGLs will be considered. A self‐reported medical diagnosis will be accepted in the recruitment of participants within studies. Studies that include but are not limited to type 1 diabetes mellitus (T1DM) will be considered to achieve the most complete data extraction on diabetes, provided that the study differentiates the data and conclusions to reflect the different management strategies of these categories. Studies that only include T1DM participants will not be considered. Studies that focus primarily on gestation diabetes (GD) will be excluded from this scoping review, despite the elevated risk of developing T2DM within their lifetime.^25^ This exclusion is due to the temporary nature of GD and the unlikely chance of returning results on participants with GD within the chosen context. There will be no exclusion of studies based on geographic location, demographic factors or socioeconomic status of participants.

### 2.3 Concept

This scoping review will consider studies that discuss interventions targeting dietary behaviors, dietary knowledge, or those that aim to connect counseling of nutritional messages for the population group to oral health outcomes. Studies that include evaluation of dietary knowledge and educational interventions relating to diet will be considered to capture as much information about the delivery of the concept in the setting and how this is considered in relation to this population. Self‐reported dietary changes and observed changes such monitored BGL or hemoglobin levels and comment on PD health or oral health outcomes will be considered. Studies that only consider clinical interventions such as periodontal treatment for the population without dietary assessment, will not be considered.

### 2.4 Context

Studies that relate to services delivered by a dental practitioner, dental team member, or delivery of the concept within a dental setting, will be considered for inclusion.

### 2.5 Search strategy

The search terms were defined by key concepts diet advice, dental practitioner, diabetes and health prevention terms. A pilot search was initially run with less specific terms and adjustments made to target the population group and the context. The chosen concepts were discussed by authors and decided upon to clearly identify results of the chosen population, concept and context whilst avoiding studies that pertain to a different population or context of the dietary intervention. The search strategies for scoping reviews available on the concept and context, but with different population targets, were manually analyzed to help identify common keywords and phrases to use within the search. The reference lists of identified articles and studies that have progressed to full text stage will be manually citation searched to determine any peer‐reviewed studies that may elude the search terms.

MEDLINE, EMBASE, CINAHL, and SCOPUS databases will be used to search for appropriate studies. Table A1 in Appendices summarizes the planned search terms that will be used within the scoping review process. Only studies and articles in English will be included. There are no limitations to the publishing dates for studies considered in this review.

### 2.6 Source of evidence selection

Following the search, all identified citations will be collated and uploaded into Covidence and duplicates removed before screening. The PRISMA‐SCR framework^26^ will be followed for identification, screening, eligibility and the results of the search; the study inclusion process will be reported in full in the final scoping review and presented in a PRISMA flow diagram. Titles and abstracts will then be screened by two or more reviewers for assessment against the inclusion criteria for the review. The full text of selected citations will be assessed in detail against the inclusion criteria by two or more reviewers. Reasons for exclusion of sources of evidence at full text that do not meet the inclusion criteria will be recorded and reported in the scoping review. Any disagreements that arise between the reviewers at each stage of the selection process will be resolved through discussion, or with an additional reviewer/s.

### 2.7 Data extraction

For the studies included in the scoping review, data will be extracted by two or more reviewers using an extraction tool developed for this scoping review. A draft of the data extraction tool will include the information found in Table A2 of the Appendices and will be revised as necessary, once the available studies have been identified in the process of extracting data. Modifications will be detailed in the scoping review. This tool will help to chart the available research on delivery of dietary messages to diabetic patients in a dental setting. The reviewers will discuss findings and any disagreements to the inclusion of information will be resolved with assessment by additional reviewers and discussion. A critical appraisal of individual sources will not be conducted, in accordance with the aims and limitations of a scoping review.^24^

### 2.8 Data presentation

**Figure A1:**
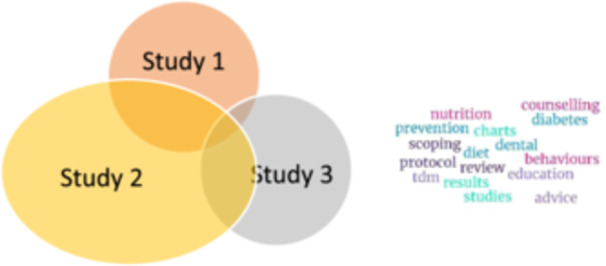
Venn diagram and Word cloud.

The data will be analyzed by quantifying text based on the methods of the studies and the discussion of the authors based on their capture of the population and the concept. The results presentation will likely be a Venn diagram and Word cloud (Figure A1 in Appendices) to visually represent similarities and differences in the study methodology, their approach to behavioral change, and the outcomes reported. This visual representation plan may be modified once the data extraction begins, and patterns or commonalities are assessed, if the studies differ substantially a Treemap may be used instead to demonstrate the individual methods of the included studies. The charts will be accompanied by a narrative summary of findings from the data and the chosen charts.

## AUTHOR CONTRIBUTIONS

**Kailey Paterson**: Conceptualization; Methodology; Writing—original draft; Writing—review and editing; Formal analysis. **Kay Franks**: Methodology; Writing—review and editing; Supervision; Conceptualization. **Janet Wallace**: Methodology; Supervision; Writing—original draft; Writing—review and editing; Conceptualization. **Dileep Sharma**: Methodology; Supervision; Project administration; Resources; Writing—review and editing. All authors have read and approved the final version of the manuscript. Dileep Sharma had full access to all of the data in this study and takes complete responsibility for the integrity of the data and the accuracy of the data analysis.

## CONFLICT OF INTEREST STATEMENT

The authors declare no conflict of interest.

## ETHICS STATEMENT

No ethical approval is needed for this review.

## TRANSPARENCY STATEMENT

The lead author Dileep Sharma affirms that this manuscript is an honest, accurate, and transparent account of the study being reported; that no important aspects of the study have been omitted; and that any discrepancies from the study as planned (and, if relevant, registered) have been explained.

## Data Availability

Data sharing is not applicable to this article as no new data were created or analyzed in this study.

## APPENDIX 1

**Table A1:** Search strategy.

*Concept 1*	*Concept 2*	*Concept 3*	*Concept 4*
diet* advice	Dental Care/or Dentists/or Oral Health/or dental profession* or Dentistry/	prevention or Primary Prevention/or Secondary Prevention/or Tertiary Prevention/	Diabetes Mellitus, Type 1/or Diabetes Mellitus/or Diabetes Complications/or Diabetes Mellitus, Type 2/or diabetes
diet* message*	Dental Hygienists/or dental hygienist*	Health Education, Dental/	
diet* counsel*	Dental Auxiliaries/or dental therapist*	Health Knowledge, Attitudes, Practice/or health knowledge or Health Education/	
diet* therapy	oral health therapist*	delivery of healthcare or “Delivery of Health Care”/	Blood Glucose/or blood glucose level.
nutrition* advice	oral health profession*	Health Promotion/or oral health promotion	hyperglycemia or Hyperglycemia/
nutrition* message*	dental practitioner*	Patient Education as Topic/or advice	diabet*
nutrition* counsel*	dental practice	counsel* or Counseling/	blood sugar or Blood Glucose/
Nutrition Therapy/or nutrition* therapy	dental team		
nutrition*			
Diet/or diet*			

**Table A2:** Extraction tool.

Identification		Population		Methodology				Context/Concept		Results and outcomes	
Reviewer	1st author + Year	Country	Participants and sample sizes	Aims of study	Study type	Data collection method	Behavioral change models?	Type of dietary interventions or assessment(s)	Setting or dental practitioner category delivering the intervention	Key outcomes as evidence of dietary counsel for diabetic patients in dental settings.	Other relevant findings
